# Decadent (?) Royalty

**Published:** 1915-01

**Authors:** 


### Decadent (?) Royalty.

It is not a direct medical issue but it is not so far from medical interest as might appear. We venture to predict, from past experience, that within six months, some prominent medical journal will print an article dealing in some way with heredity, in which allusion will be made to the influences of in-breeding, profligacy and the general effects of spoiling by wealth and flattery, so that the royal families have become an international assembly of idiots or madmen, cowards, rakes and spendthrifts. Having such statements in mind, we ask our readers to ponder over the news notes of the war and other matters of recent history, as applying to the monarchs and their immediate families in the following countries: England, Germany, Russia, Austria, Italy, Greece, Belgium, Holland, Norway and Sweden, Denmark, Spain and the Balkan States. We do not imply unanimous agreement with policies, especially regarding war, nor do we ask you to go into ecstacies over the human excellences to be expected of men generally, nor to overlook human deficiencies. We are not advocating anything contrary to American principles. But, as a matter of fairness, we think it must be conceded that the royal families of Europe have utterly failed to serve as the warning examples which a pessimistic class of orators and authors have held them to be. That there have been unworthy individuals in this large number of more or less related families, can not be denied but, on the whole, they cannot be regarded as decadent as compared with any analogous group of monarchic families at any period in the world's history. And, so far as we can judge, no similar group of families, related and connected in approximately the same ways, taken from any walk of life, in this country, or in any other, would show more nobility of character, intelligence, courage or judgement; or would even rank higher in purely physical development and longevity.

